# Efficacy of Taiji Stick exercise on sleep quality and anxiety in older adults: a randomized controlled trial

**DOI:** 10.3389/fpsyg.2026.1760046

**Published:** 2026-02-20

**Authors:** Longfei Cao, Xiaoxiao Dong, Kai Qi, Chunhui Zhou, Aiguo Chen

**Affiliations:** 1 Gdansk University of Physical Education and Sport, Gdansk, Poland; 2 Jiangsu Vocational Institute of Commerce, Nanjing, Jiangsu, China; 3 Nanjing Sport Institute, Nanjing, Jiangsu, China; 4 Soochow University, Suzhou, Jiangsu, China

**Keywords:** anxiety, older adults, senior care center, sleep quality, Taiji Stick

## Abstract

**Introduction:**

Existing studies have confirmed that unarmed Health Qigong exercise can effectively improve sleep quality and reduce anxiety level among older adults; however, research on the effects of equipment-based Health Qigong practice on sleep quality and anxiety level in older adults remains scarce. Thus, this research sought to examine how Taiji Stick training over 11 weeks affects sleep quality and anxiety in older individuals.

**Methods:**

This study employed a 2 × 2 mixed randomized controlled design, 35 senior participants were randomly divided into an experimental group (*n* = 17) and a control group (*n* = 18). The experimental group underwent an intervention based on Taiji Stick practice with a 11-week duration, three sessions were conducted each week, with each session lasting 45 min, while the control group received no intervention and maintained their usual lifestyle habits. Sleep quality and anxiety level among older population in the both groups were measured using the Zung Self-Rating Anxiety Scale (SAS) and the Pittsburgh Sleep Quality Index (PSQI) before and after exercise intervention.

**Results:**

In comparison to the pre-test findings within the experimental group, The post-test results showed a significant decline in the PSQI global score (*P* < 0.001), and scores of the four components in the PSQI, including subjective sleep quality (*P* < 0.05), sleep disturbances (*P* < 0.01), use of sleeping medication (*P* < 0.01), and daytime dysfunction (*P* < 0.05), were all significantly reduced. Meanwhile, the experimental group demonstrated a marked decrease in their post-test SAS standard score (*P* < 0.01). A notable rise was observed in the control group's post-test PSQI global score compared to the pre-test (*P* < 0.01), and the sleep efficiency component score in PSQI also significantly increased (*P* < 0.01). Additionally, the control group exhibited a marked increase in their post-test SAS standard score (*P* < 0.001). Following the exercise intervention, significant reductions were observed on both the PSQI global score and SAS standard score of the experimental group compared with the control group (PSQI global score: *P* < 0.001; SAS standard score: *P* < 0.001).

**Conclusion:**

This study's findings revealed that 11 weeks of Taiji Stick exercise notably enhanced sleep quality and reduced anxiety level in older individuals.

## Introduction

With the increasing aggravation of the global aging trend, the number of older adults continues to grow. As projected by the United Nations, the global population of older adults aged 65 and over is anticipated to hit 1.6 billion by 2050, occupying approximately one-fifth of the global total population (Koldaş, 2017; Naseer et al., 2025). As people age and their living environment changes, older adults often face multiple physical and mental health challenges. Besides the gradual decline of physical functions, older adults might also encounter a deterioration in sleep quality (Gulia and Kumar, 2018). Moreover, there is a heightened probability of developing emotional disorders like anxiety (Riadi et al., 2020).

A systematic review and meta-analysis indicated that roughly 40% of older adults living in communities worldwide experience suboptimal sleep quality (Canever et al., 2024). Of particular note, among older adults in Chinese care homes, this proportion stands at 67.3% (Zhu et al., 2020). Long-term sleep deprivation is known to compromise the living quality of older adults, but may also trigger systemic low-grade chronic inflammation, thereby inducing various inflammation-related chronic diseases For instance, diabetes, atherosclerosis, Alzheimer's disease (Besedovsky et al., 2019; Hurtado-Alvarado et al., 2013). Previous research findings have indicated that the mid-to-older adults with sleep disorders have a 1.89-fold higher likelihood of anxiety development vs. their non-sleep-disordered counterparts. Likewise, older adults with anxiety had a 1.20-fold greater likelihood of sleep disorder development compared with their non-anxious counterparts. Thus, there is a bidirectional association between the two, in which they mutually influence each other (Peng et al., 2024; Spira et al., 2009). The common mental health issue of anxiety cannot be ignored in older adults. Globally, the overall incidence of anxiety in older adults was approximately 16.5% (Jalali et al., 2024), with further research indicating that an annual incidence of anxiety among older adults aged 65 and above can even reach 20.8% (Cisneros and Ausín, 2019). Although anxiety is relatively common among older adults, its potential harm is often underestimated. Chronic anxiety not only significantly affects the emotional health of older adults, but may also have adverse impacts on their cognitive and physiological functions. Prior studies have shown that chronic anxiety may elevate the risk of developing various diseases, including cancer, cardiovascular diseases and dementia (Lenze and Wetherell, 2011; Andreescu and Lee, 2020). Therefore, to enhance sleep quality and alleviate anxiety level among older adults is of great significance for promoting their physiological and psychological well-being alongside life quality.

The common intervention methods for improving sleep quality in older adults mainly include drug therapy, exercise intervention, and cognitive-behavioral intervention. Among them, although drug therapy can rapidly relieve sleep problems in the short term, long-term use is often accompanied by certain risks, such as an increase in the incidence of fall-induced trauma, a decline in cognitive function, and a heightened risk of dementia development (Treves et al., 2018; Paterniti et al., 2002; He et al., 2019). Therefore, an increasing number of researchers and clinical workers have turned their attention to non-drug therapies for enhancing sleep quality among older adults. As the primary non-drug intervention approaches for improving sleep quality among older adults, exercise intervention and cognitive-behavioral intervention demonstrate equivalent efficacy. However, further comparison reveals that exercise intervention has more advantages in terms of implementation convenience and large-scale group intervention. Thus, research focusing on non-drug group interventions to enhance sleep quality among older adults often prioritizes exercise-based approaches. At present, the typical exercise-based interventions applied to improve sleep quality among older adults primarily include Baduanjin, Wuqinxi, Tai Chi (Chou et al., 2024; Vanderlinden et al., 2020; Khaleghi and Ahmadi, 2024).

In terms of reducing the anxiety level of older adults, commonly adopted intervention measures include drug therapy, social support, psychological intervention and exercise intervention. Although pharmacological therapy takes effect relatively quickly, long-term use may lead to obvious side effects, such as fatigue, somnolence, cognitive function decline, increased risk of falls, and urinary system diseases (Lenze et al., 2009; Madhusoodanan and Bogunovic, 2004). Therefore, it needs to be used with caution. Previous studies have shown a notable inverse relationship linking social support received by older adults and their degree of anxiety (Leung et al., 2007; Zhao et al., 2022). However, in real life, older adults often face the dilemma of gradually decreasing social support resources. The main reasons include reduced companionship from children, widowhood or living alone, weakening of social roles after retirement, and mobility limitations caused by physical function degradation. Since these influencing factors are constrained by objective conditions, they are usually difficult to be fundamentally resolved. Therefore, it is necessary to seek other alternative intervention measures to make up for the insufficiency of social support. A systematic review and meta-analysis pointed out that active participation in physical exercise not only helps improve individuals' physiological and psychological wellness, but also enhances the sense of subjective social support by promoting social interaction and communication, thereby effectively alleviating the anxiety of older adults (Sulandari et al., 2024). Additionally, exercise intervention has more advantages than psychological intervention in terms of implementation convenience and large-scale group intervention. Hence, researches on non-drug group interventions for anxiety reduction among older adults tend to prefer exercise interventions. It has been demonstrated that exercise programs like Tai Chi, Baduanjin, and Liuzijue serve as intervention measures to reduce anxiety level in older adults, achieving remarkable effects (Wu et al., 2022; Feng et al., 2025).

Compared with the risk of adverse effects of pharmacotherapy and the predicament of limited accessibility to social support resources, Chinese traditional sport interventions feature low economic cost, minimal safety risks and feasibility for group implementation, and can enhance older adults' subjective social support and social participation by facilitating social interactions. Taiji Stick is currently the only implement-based form among the 11 Health Qigong drills promoted by the (General Administration of Sport of China. Health Qigong, 2020).^1^ It shares the same evolutionary origin and bears a high degree of similarity in movement patterns with such exercises as Baduanjin and Wuqinxi, laying a solid theoretical and practical foundation for this study. Based on this, we hypothesize that Taiji Stick exercise can improve sleep quality and reduce anxiety level in older adults, and for the first time adopt a randomized controlled design to explore its intervention effects. Thus, this study fills the research gap regarding the effects of implement-based Health Qigong intervention on sleep quality and anxiety level in older adults.

## Methods

### Study design and registration

A 2 × 2 mixed-factorial design was adopted, with factors including Group (intervention vs. control) and Time (before intervention vs. after intervention), Group functioned as a between-participants factor while Time served as a within-participants factor. The experimental protocol was divided into three sequential stages: an initial evaluation period, an intervention based on Taiji Stick training spanning 11 weeks, and an immediate evaluation after the intervention. The study was conducted at the Zhongshan-Meiyuan senior care center in China's Jiangsu Province, Nanjing City, between February and May 2025. This study secured ethical clearance from Nanjing Sport Institute's Human Research Ethics Committee (Ethics Clearance No. RT-2025-04) and adhered to the guidelines of the Helsinki Declaration. All participants provided written informed consent. Additionally, this research was authorized for registration by the Chinese Clinical Trial Registry (ChiCTR2400083424) before participant enrollment.

### Participants

Researcher calculated the required sample size using G^*^Power 3.1 software, specifically for an analysis of variance with repeated measures (*Es* = 0.25, α = 0.05, Power = 0.80), according to the calculation, no fewer than 34 older adults are required. Older adults were recruited based on predefined exclusion and inclusion criteria. The eligibility criteria were as follows: (1) participants aged 65 years and over, irrespective of gender; (2) participants with unimpaired limbs; (3) those with an educational attainment of junior high school or higher; (4) participants without previous exposure to Taiji stick exercise; and (5) participation on a voluntary basis, confirmed by the signing of informed consent documents. The exclusion criteria were specified below: (1) participants with severe psychological or physical disorders; (2) participants experiencing significant vision impairment; (3) participants incapable of standing or moving without supportive equipment; and (4) Those who have experienced lower limb fracture over the past half-year. Participants were excluded from the ultimate analytical process if they abandoned their involvement in the study prior to it being completed, or missed three sequential sessions or accrued six absences in total throughout the intervention period.

**Figure 1:**
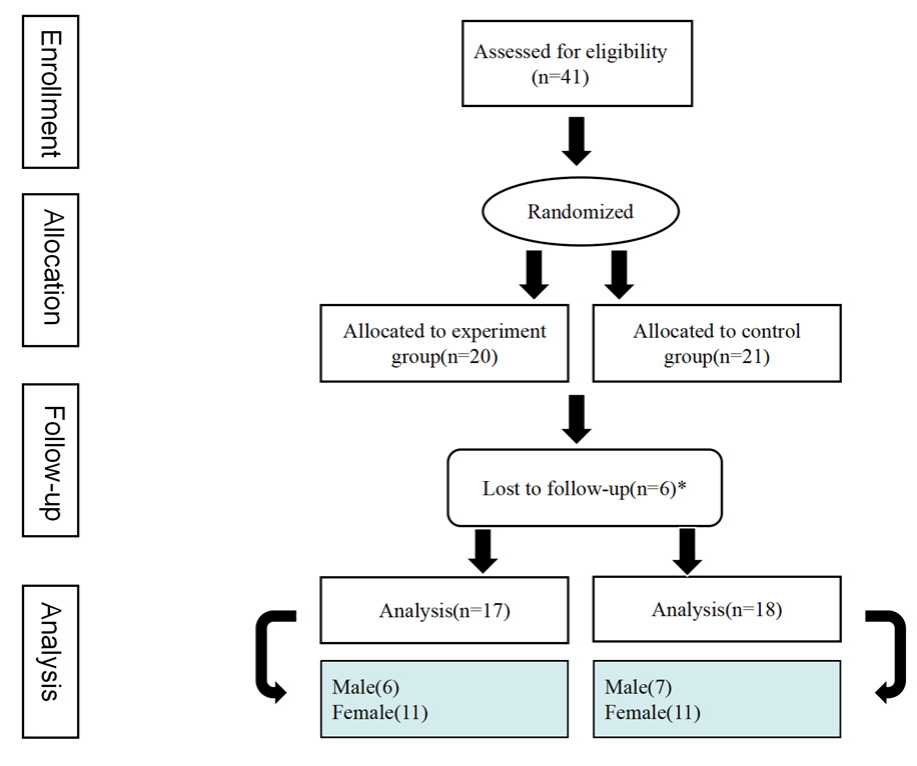
Participant flow chart.

Forty-one older adults were enrolled from a senior care center in Nanjing for this study. Prior to randomization, we divided participants into two groups by gender: the male group (15 participants) and the female group (26 participants). Two opaque boxes were prepared, labeled Box A and Box B respectively, with a hand-accessible circular hole on the top of each box. Box A contained 15 cards, each marked with a unique number ranging from 1 to 15; Box B held 26 cards, each marked with a unique number ranging from 1 to 26. Each male participant randomly drew a card from Box A, and each female participant randomly drew a card from Box B, with the drawn card serving as their respective unique identification number. Subsequently, two identical sealed opaque envelopes were prepared by a senior care center staff not involved in the study, each containing a different grouping rule: one stated that “odd numbers go to the experimental group and even numbers go to the control group”; the other specified that “even numbers go to the experimental group and odd numbers go to the control group”. Finally, a Taiji Stick instructor who did not know any of the participants randomly selected one envelope, opened it on-site, and the final group assignment was completed in accordance with the rule contained in the envelope. Ultimately, the intervention group consisted of 20 participants: 13 females and seven males, the control group included 21 participants: 13 females and eight males. Three participants from each group withdrew during the intervention. This study reported a participant attrition rate of 14.63%, which stood below the widely recognized 20% (Cramer et al., 2016). As a result, data from 35 participants was incorporated into the conclusive analysis. the intervention group was comprised of 17 participants, including six males and 11 females; whereas the control group was composed of 18 participants, including seven males and 11 females (Figure 1). In this research, the intervention group exhibited a compliance rate of 95.72%, with an average of 31.59 session attendances.

### Intervention program

**Figure 2:**
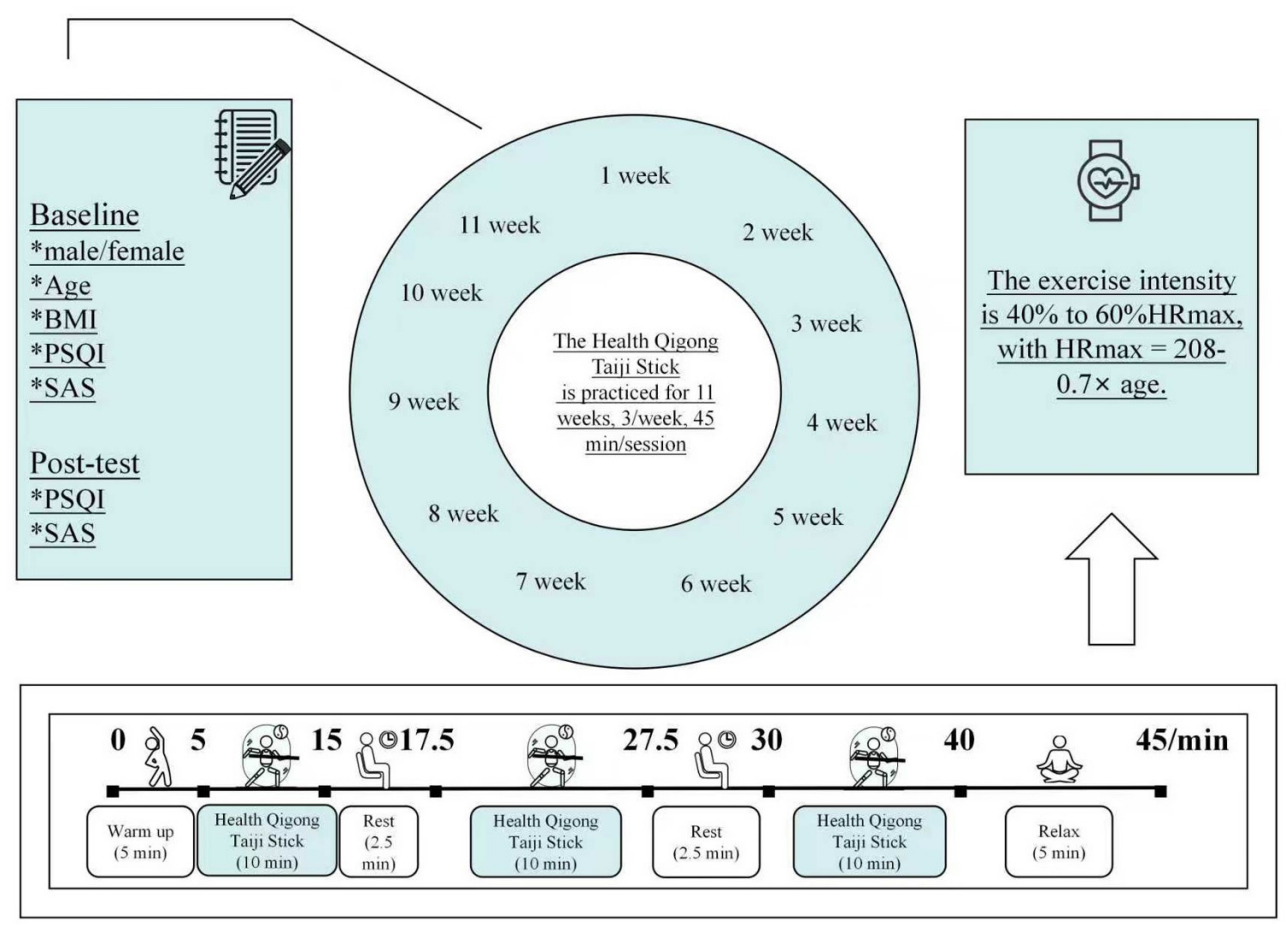
Each session intervention procedure.

Taiji Sticks are made of wood, and each has an approximate weight of 0.42 kg. Taiji Stick practice served as the intervention measure. Participants in this study were taught Taiji Stick by the same certified instructor in all practice sessions, who graduated with a major in Traditional Chinese Exercises from a sport university and has 15 years of teaching experience in Traditional Chinese Exercises. The intervention group practiced for a total of 11 weeks, three sessions per week, with each session lasting 45 min. Each week, on Monday, Wednesday, and Friday, the intervention group engaged in training sessions at 9:00–9:45 a.m. Each 45-min session included: 5 min preparatory exercise, 10 min of practicing the Taiji Stick, 2.5 min rest interval, subsequent 10 min of practicing the Taiji Stick, a follow-up rest period lasting 2.5 min, 10 min of practicing the Taiji Stick for the third time, a final 5-min wind-down exercise to close the session (Figure 2). The heart rates of two participants were measured in sequence for each session, and the exercise intensity for each session was assessed by the immediate post-exercise heart rate. For the specific measurement procedure, After the end of each session, two professional nursing staff immediately palpated the radial artery on the right wrist of the two participants, counted the pulse for 10 s, multiplied the count by six, and obtained each participant's immediate post-exercise heart rate (beats per minute, bpm). A 40%−60% HRmax range was used for the exercise intensity of each session, and HRmax is computed as 208 – 0.7 × age (Tanaka et al., 2001). Table 1 sets out detailed weekly and general instruction schemes for Taiji Stick practice.

**Table 1:** Taiji Stick training arrangement.

**Week**	Weekly exercise schedule		
	**Monday program**	**Wednesday program**	**Friday program**
1	The left style in form 1 (Shaogong Yaolu)	The right style in form 1 (Shaogong Yaolu)	The complete style of form 1 (Shaogong Yaolu)
2	The left style in form 2 (Qingzhou Huanxing)	The right style in form 2 (Qingzhou Huanxing)	The complete style of form 2 (Qingzhou Huanxing)
3	The left style in form 3 (Fengbai Heye)	The right style in form 3 (Fengbai Heye)	The complete style of form 3 (Fengbai Heye)
4	The left style in form 4 (Chuanfu Beiqian)	The right style in form 4 (Chuanfu Beiqian)	The complete style of form 4 (Chuanfu Beiqian)
5	The left style in form 5 (Shenzhen Dinghai)	The right style in form 5 (Shenzhen Dinghai)	The complete style of form 5 (Shenzhen Dinghai)
6	The left style in form 6 (Jinlong Jiaowei)	The right style in form 6 (Jinlong Jiaowei)	The complete style of form 6 (Jinlong Jiaowei)
7	The left style in form 7 (Tanhai Xunbao)	The right style in form 7 (Tanhai Xunbao)	The complete style in form 7 (Tanhai Xunbao)
8	Form 8 (Qigui Dantian) closing posture	Form 8 (Qigui Dantian) closing posture	Form 8 (Qigui Dantian) closing posture
9	Reinforce form 1	Reinforce forms 1–2	Reinforce forms 1–3
10	Reinforce forms 1–4	Reinforce forms 1–5	Reinforce forms 1–6
11	Reinforce forms 1–7	Reinforce forms 1–8	Reinforce forms 1–8 and closing posture

## Outcome measures

### Sleep quality

The subjective quality of sleep of the study participants was assessed using the Pittsburgh Sleep Quality Index (PSQI). This scale is made up of 19 items that require self-rating and five items that need to be rated by observer. Among these self-assessed items, the 18 self-rated items involved in scoring form seven components, which include: subjective quality of sleep, sleep onset latency, duration of sleep, efficiency of sleep, disturbances during sleep, utilization of sleeping drugs, daytime functional impairment. Each of the components is rated using a scale ranging from 0 to 3, with the cumulative score varying between 0 and 21. An increased score signifies worse sleep quality in the subjects (Buysse et al., 1989).

### Anxiety level

The Zung Self-Rating Anxiety Scale (SAS) served to assess the level of anxiety as subjectively perceived by the study participants. The scale includes 20 self-rated items, of which 15 are positively scored items, each rated on a 1–4 scale; there are 5 reverse-scored items (Canever et al., 2024; Peng et al., 2024; Lenze and Wetherell, 2011; He et al., 2019; Vanderlinden et al., 2020), each scored on a 4–1 scale. The rating criteria are as follows: A rating of 1 represents absence of time or very little time, a rating of two represents a small share of the time, a rating of three represents a notable portion of time, and a rating of 4 represents nearly all or all of the time. Subsequently, summing all 20 items' scores yields the initial total score, which ranges from 20 to 80 points. The original score first undergoes multiplication by 1.25, and then the integral part of the result is extracted to obtain the standard score, which ranges from 25 to 100 points (Zung, 1971). Based on the findings of the norm for the Chinese population, the SAS standard score uses 50 points as its cut-off value, and this value functions as the benchmark for evaluating anxiety level. In detail, a score range of 50–59 represents slight anxiety, 60–69 denotes medium anxiety, and any score exceeding 70 indicates severe anxiety (Zhang et al., 2021; Gao et al., 2023).

### Statistical analysis

SPSS 27.0 was used to carry out statistical analyses. Continuous variables' normality was examined before proceeding with subsequent analytical operations. Mean ± standard deviation (Mean ± SD) was used to presented the continuous variables. The non-parametric Mann–Whitney U test was applied to baseline data that deviated from a normal distribution. By contrast, the independent samples *t*-test was adopted for baseline data that followed a normal distribution. Additionally, the chi-square test was employed to contrast the gender distribution between the two groups, all aiming to assess if their baseline characteristics were homogeneous. Normal-distributed data were analyzed using the repeated measures Analysis of Variance (ANOVA) to explore variations of indicators in the two groups pre- and post-intervention. The analysis strategy was determined by the time × group interaction result. Specifically, main effect analysis was performed when the interaction did not show significance, whereas simple effect analysis was employed when the interaction reached significance. Bonferroni correction was applied to conduct multiple *post-hoc* pairwise comparisons. Greenhouse–Geisser correction procedure came into use in instances where the sphericity test failed to reach satisfaction. In cases where data deviated from the normal distribution, Wilcoxon signed-rank test (a non-parametric method) came into application. Finally, statistical significance was signified by a *P*-value under 0.05.

## Results

### Baseline homogeneity test

Before the exercise intervention, homogeneity tests were performed on BMI (kg/m^2^), age (years old), gender (male/female), the standard score of the SAS, as well as the comprehensive score and each dimension's score of the PSQI within the two groups. The results demonstrated no significant differences, indicating that the two groups shared homogeneous baseline characteristics (Table 2).

**Table 2:** Baseline characteristics (mean ± SD)/median (Q1, Q3).

**Variables**	**Intervention group**	**Control group**	**χ^2^**	***T*/*Z***	***P***
*N*	17	18	–	–	–
Gender (male/female)	6/11	7/11	0.048	–	0.826
Age (years)	83.71 ± 2.89	85.22 ± 2.78	–	−1.583	0.123
BMI (kg/m^2^)	23.20 ± 2.99	24.18 ± 3.31	–	−0.922	0.363
PSQI (global score)	8.94 ± 3.83	8.83 ± 3.59	–	0.086	0.932
SAS (standard score)	43.29 ± 5.73	45.17 ± 6.84	–	−0.875	0.388
Subjective sleep quality	1 (1, 1.5)	1 (1, 2)	–	−0.079	0.937
Sleep latency	1 (1, 2)	2 (1, 3)	–	−0.910	0.363
Sleep duration	1 (0, 2)	0 (0, 1.25)	–	−0.838	0.402
Sleep efficiency	0 (0, 1)	0 (0, 1)	–	−0.290	0.772
Sleep disturbances	2 (1.5, 2)	2 (1.75, 2)	–	−0.091	0.928
Sleeping medication use	1 (0, 3)	1 (0, 3)	–	−0.017	0.986
Daytime dysfunction	2 (1, 2)	2 (1, 2)	–	−0.240	0.810

PSQI, Pittsburgh sleep quality index; SAS, self-rating anxiety scale.

### Sleep quality changes: between and within two groups pre- and post-intervention

#### PSQI global score

The repeated measures ANOVA revealed no statistical significance for the main effect of time [*F*_(1, 33)_ = 2.071, *P* = 0.160, $\eta_{p}^{2}$ = 0.059] or group [*F*_(1, 33)_ = 3.440, *P* = 0.073, $\eta_{p}^{2}$ = 0.094]; in contrast, the interactive relationship reflected statistical significance between time and group [*F*_(1, 33)_ = 30.149, *P* < 0.001, $\eta_{p}^{2}$ = 0.477]. Subsequent simple effect analysis revealed that the pre-test PSQI global scores did not differ significantly between the two groups [*F*_(1, 33)_ = 0.007, *P* = 0.932 > 0.05, $\eta_{\text{p}}^{2}$ = 0.000], whereas a notable difference was observed in the post-test global scores of the PSQI [*F*_(1, 33)_ = 21.573, *P* < 0.001, $\eta_{\text{p}}^{2}$ = 0.395]; A significance at the statistical level in the PSQI global scores was found between the pre-test and post-test within the control group [*F*_(1, 33)_ = 8.449, *P* = 0.006 < 0.01, $\eta_{p}^{2}$ = 0.204], with the post-test mean exceeding the pre-test mean; A statistically noteworthy variation in the PSQI global scores was observed for the experimental group between pre-test and post-test [*F*_(1, 33)_ = 23.345, *P* < 0.001, $\eta_{p}^{2}$ = 0.414], while the post-test mean was lower than the pre-test mean. A marked positive impact of Taiji Stick exercise on the overall sleep quality of the elderly was supported by these results (Table 3).

**Table 3:** Results of ANOVA for repeated measurements (mean ± SD).

**Variables**	Intervention group (***n*** = 17)		Control group (***n*** = 18)	
	**Pretest**	**Posttest**	**Pretest**	**Posttest**
PSQI (global score)	8.94 ± 3.83	6.47 ± 1.84^bd^	8.83 ± 3.59	10.28 ± 2.87^b^
SAS (standard score)	43.29 ± 5.73	38.71 ± 3.92^bd^	45.17 ± 6.84	51.94 ± 5.41^b^

Posttest compared to pretest within the same group: ^a^(*P* < 0.05), ^b^(*P* < 0.01).

Comparison between groups at the same time point: ^c^(*P* < 0.05), ^d^(*P* < 0.01).

#### PSQI component score

Wilcoxon signed-rank test confirmed that the experimental group presented notable decreases in post-test vs. pre-test scores for the PSQI components listed below: Subjective sleep quality (*Z* = −2.070, *P* = 0.038 < 0.05, |*r*| = 0.926); Sleep disturbances (*Z* = −3.317, *P* = 0.001 < 0.01, |*r*| = 0.999); Use of sleeping medication (*Z* = −2.810, *P* = 0.005 < 0.01, |*r*| = 0.937); Daytime dysfunction (*Z* = −2.111, *P* = 0.035 < 0.05, |*r*| = 0.746). Conversely, the experimental group exhibited no significant score changes across the following PSQI components when comparing test results before and after the intervention: Sleep latency (*Z* = −1.069, *P* = 0.285 > 0.05, |*r*| = 0.322); Sleep duration (*Z* = −1.000, *P* = 0.317 > 0.05, |*r*| = 0.408); Sleep efficiency (*Z* = −0.176, *P* = 0.860 > 0.05, |*r*| = 0.066). The Wilcoxon signed-rank test showed no significant changes in post-test vs. pre-test scores for the following PSQI components in the control group: Subjective sleep quality (*Z* = −1.000, *P* = 0.317 > 0.05, |*r*| = 0.500); Sleep latency (*Z* = −1.027, *P* = 0.305 > 0.05, |*r*| = 0.325); Sleep duration (*Z* = −0.333, *P* = 0.739 > 0.05, |*r*| = 0.136); Sleep disturbances (*Z* = −0.378, *P* = 0.705 > 0.05, |*r*| = 0.143); Use of sleeping medication (*Z* = −1.000, *P* = 0.317 > 0.05, |*r*| = 0.408); Daytime dysfunction (*Z* = −1.890, *P* = 0.059 > 0.05, |*r*| = 0.714). However, when looking at the control group from pre-test to post-test, a substantial rise in the sleep efficiency scores was observed (*Z* = −2.652, *P* = 0.008 < 0.01, |*r*| = 0.799). It was suggested by these results that older adults' Subjective sleep quality, Sleep disturbances, Use of sleeping medication, and Daytime dysfunction were markedly improved through Taiji Stick exercise (Table 4).

**Table 4:** Results of Wilcoxon signed rank test median (Q1, Q3).

**PSQI (component score)**	Intervention group (***n*** = 17)		Control group (***n*** = 18)	
	**Pretest**	**Posttest**	**Pretest**	**Posttest**
Subjective sleep quality	1 (1, 1.5)	1 (1, 1)^a^	1 (1, 2)	1 (1, 2)
Sleep latency	1 (1, 2)	1 (1, 1.5)	2 (1, 3)	2 (1, 3)
Sleep duration	1 (0, 2)	1 (0, 1)	0 (0, 1.25)	0.5 (0, 1)
Sleep efficiency	0 (0, 1)	0 (0, 1)	0 (0, 1)	1 (1, 1)^b^
Sleep disturbances	2 (1.5, 2)	1 (1, 1)^b^	2 (1.75, 2)	2 (1, 2)
Sleeping medication use	1 (0, 3)	0 (0, 1)^b^	1 (0, 3)	1 (0.75, 3)
Daytime dysfunction	2 (1, 2)	1 (1, 2)^a^	2 (1, 2)	2 (1, 2.25)

Posttest compared to pretest within the same group: ^a^(*P* < 0.05), ^b^(*P* < 0.01).

#### SAS standard score

The repeated measures ANOVA demonstrated no statistical significance for the main effect of time [*F*_(1, 33)_ = 1.236, *P* = 0.274, $\eta_{p}^{2}$ = 0.036]. In contrast, both the main effect of group [*F*_(1, 33)_ = 21.918, *P* < 0.001, $\eta_{p}^{2}=$ 0.399] and the time × group interaction effect [*F*_(1, 33)_ = 33.302, *P* < 0.001, $\eta_{p}^{2}$ = 0.502] exhibited the statistical significance. Further analysis of simple effects revealed that the pre-test SAS standard scores did not differ significantly with respect to the experimental vs. control groups [*F*_(1, 33)_ = 0.766, *P* = 0.388 > 0.05, $\eta_{p}^{2}$ = 0.023], however, in terms of post-test SAS standard scores, a pronounced difference existed as regards the two groups [*F*_(1, 33)_ = 68.103, *P* < 0.001, $\eta_{p}^{2}$ = 0.674]; The pre-test and post-test SAS standard scores of the control group exhibited a marked difference [*F*_(1, 33)_ = 24.380, *P* < 0.001, $\eta_{p}^{2}$ = 0.425], and the mean obtained in the follow-up measurement exceeded that of the baseline measurement; A significant difference in SAS standard scores between the pre-test and post-test was detected in the experimental group [*F*_(1, 33)_ = 10.552, *P* = 0.003 < 0.01, $\eta_{p}^{2}$ = 0.242], whereas the mean from the post-test was lower in comparison to that from the pre-test. As indicated by the above results, Taiji Stick exercise could significantly lessen the anxiety level of older adults (Table 3).

#### Safety assessment

There were no adverse events associated with Taiji Stick exercise throughout the study period.

## Discussion

Taiji Stick is a Chinese traditional sport that uses the wooden stick as a guide to channel “qi” flow, integrates bodily forms with the circulation of “qi”, and unifies forms and spirit. In the practice of Taiji Stick, it emphasizes the waist as the axis, focuses on coordinated whole-body movements such as twisting, rotating, bending, and stretching, and synergizes with internal activities including breathing, mental intention, and verve to achieve mind-body unity. This study found that older adults who engaged in 11 weeks of Taiji Stick exercise training (three 45-min sessions per week) experienced significant improvement in sleep quality and reduction in anxiety level, thereby confirming the hypothesis proposed in this research.

After an 11-week Taiji Stick exercise intervention, marked improvements in overall sleep quality, subjective quality of sleep, disturbances during sleep, utilization of sleeping drugs, and daytime functional impairment were detected among older participants in the experimental group. The results of this study generally aligned with earlier research findings on Tai Chi, Baduanjin, and Qigong interventions. A previous study has shown that older women with knee osteoarthritis who engaged in 60-min Tai Chi session, three sessions weekly for a 24-week period, exhibited enhancements with respect to overall sleep quality as well as in terms of alleviating sleep disturbances (Lü et al., 2017). Another study confirmed that community-residing older adults who performed Baduanjin training three sessions a week, a 45-min duration per training session, over 16 weeks, showed positive changes in overall sleep quality, subjective sleep experience, and reduced daytime dysfunction (Chen et al., 2012). Additionally, results derived from a meta-analysis indicated that Qigong training has a positive effect on reducing disturbances in sleep among older individuals (Makhfudli et al., 2024). Given the effects achieved by the above exercise intervention, therefore, investigating the mechanisms that enable the above exercise to enhance sleep quality in older adults is necessary. Relevant studies have indicated that practicing Tai Chi or Baduanjin can enhance parasympathetic nerve tension, reduce sympathetic nervous system activity, and thereby alleviating physiological hyperarousal. Such exercise intervention can also reduce pro-inflammatory cytokines over a prolonged period, raise the levels of neurotrophic factors derived from the brain, and reinforce the brain's capacity for neuroplasticity as well as circadian rhythm regulation. Furthermore, the aforementioned exercise interventions serve to optimize melatonin secretion, a vital hormone responsible for regulating sleep-wake rhythms (Bu et al., 2025; Li et al., 2024; Fan et al., 2020).

After undergoing an 11-week intervention of Taiji Stick exercise, the experimental group's older participants experienced a marked reduction in anxiety, and this outcome was roughly in line with previous research findings on interventions including Tai Chi, Baduanjin, and Qigong. Related meta-analyses along with reviews based on systematic literatures have pointed out that after the elderly performed Tai Chi, Qigong, or Baduanjin exercises for 20–30 min each session, 2–5 sessions per week over 10–24 weeks, their anxiety state was significantly improved (Kuang et al., 2024; Jones et al., 2022; Dong et al., 2025). A study has indicated that Tai Chi or Qigong practice exerts an impact on emotional regulation. This effect could be achieved by altering several regions including prefrontal areas, the limbic network, as well as the striatal complex, alternatively through gene activity pertaining to inflammatory reactions in addition to stress-linked routes (Yeung et al., 2018). Another study has found that practicing Tai Chi can lead to an increase in brain theta oscillatory power and reduce anxiety levels (Wang et al., 2024). In recent years, the advancements in neuroimaging techniques have offered novel research perspectives for revealing how Tai Chi practice influences different brain regions' anatomical morphology alongside their neural activities (Yu et al., 2018).

In summary, Taiji Stick, Baduanjin, Qigong, and Tai Chi are all classified as traditional sports of China, with their exercise characteristics (low-intensity, slow movements) as well as exercise concepts (integrated cultivation of both internal and external aspects, unity of body and mind) are also basically consistent (Li et al., 2020; Wang and Luo, 2024; Cao et al., 2025). From the above, this study inferred that the mechanisms by which Taiji Stick practice improved sleep status and anxiety level among older individuals might align with the mechanisms underpinning the positive impacts of Qigong, Baduanjin, and Tai Chi exercises on the same outcomes.

While this study has yielded worthwhile achievements, several limitations remain. First, the analysis included merely 35 participants at the end of the intervention, resulting in A limited sample size might compromise the sample's representativeness along with research findings' stability. Second, as the elderly required to be informed of the research purpose during recruitment, it was impossible to implement blinding for the elderly subjects in this study. Therefore, certain biases might have been introduced into the measurement results when evaluating the indicators for the participants. To minimize the subjective evaluation bias caused by the evaluators' expectancy effect as much as possible, this study implemented a blinding method for the evaluators during the processes in the scales' pre-assessment and post-assessment. The specific measures were as follows: All evaluators were professional nursing staff from the senior care center, who had no knowledge of this study's purpose nor of how participants were grouped. The scale assessments for the participants in this study were administered by four nursing staff following the daily work procedures. Moreover, the Taiji Stick intervention's long-term sustained effects within a certain period after the intervention ceased lacked an exploration in this study. This study was initially designed to verify whether elderly individuals' sleep quality and anxiety status could be ameliorated through Taiji Stick intervention (short-term effects), so as to lay a foundation for subsequent long-term follow-up study. In existing studies on Tai Chi intervention, most studies only evaluated the immediate effects when the intervention was completed. In the event that an extra follow-up procedure was implemented, the predominant follow-up time point was 3 months after the completion of the intervention (Carbonell-Baeza et al., 2011; Wang et al., 2010). Guided by this, we intend to carry out a follow-on evaluation 3 months after the intervention concludes within future studies on Taiji Stick exercise, besides assessing short-term impacts from the intervention. This measure aims to explore the maintenance of the intervention effects and in turn uncover the practical worth derived from Taiji Stick training in greater depth.

Based on the findings of this study, subsequent controlled trials of Taiji Stick vs. other traditional Chinese exercises (such as Wuqinxi, Baduanjin) are to be conducted, aiming to clarify which of these practices yields superior intervention effects among older adults with respect to sleep quality as well as anxiety level. Additionally, further studies will explore the physiological, biological, neuroscientific, and brain science mechanisms underlying the impacts of Taiji Stick practice concerning the quality of sleep as well as the level of anxiety among older adults. This line of study aims to comprehensively elucidate the scientific basis of Taiji Stick exercise effects.

## Conclusion

This research's results demonstrated that after partaking in a Taiji Stick training program spanning 11 weeks, older adults exhibited substantial enhancement in sleep quality alongside reduced anxiety level. These results provided essential empirical evidence to support the future promotion and popularization of Taiji Stick practice.

## Data Availability

The original contributions presented in the study are included in the article/supplementary material, further inquiries can be directed to the corresponding author.

## Footnotes

1 https://www.sport.gov.cn/qgzx/n5402/c957402/content.html (Accessed April 5, 2025).
